# Modulation of actin dynamics as potential macrophage subtype-targeting anti-tumour strategy

**DOI:** 10.1038/srep41434

**Published:** 2017-01-30

**Authors:** Carlo Pergola, Katrin Schubert, Simona Pace, Jana Ziereisen, Felix Nikels, Olga Scherer, Stephan Hüttel, Stefan Zahler, Angelika M. Vollmar, Christina Weinigel, Silke Rummler, Rolf Müller, Martin Raasch, Alexander Mosig, Andreas Koeberle, Oliver Werz

**Affiliations:** 1 Chair of Pharmaceutical/Medicinal Chemistry, Institute of Pharmacy, Friedrich-Schiller-University, Jena, Germany; 2 Helmholtz-Institute for Pharmaceutical Research Saarland (HIPS), Saarbrücken, Germany; 3 Helmholtz Centre for Infection Research and Pharmaceutical Biotechnology at Saarland University, Saarbrücken, Germany; 4 Department of Pharmacy, Pharmaceutical Biology, Ludwig-Maximilians-University, 81377 Munich, Germany; 5 Institute of Transfusion Medicine, University Hospital Jena, Jena, Germany; 6 Institute of Biochemistry II, University Hospital Jena, Jena, Germany.

## Abstract

Tumour-associated macrophages mainly comprise immunosuppressive M2 phenotypes that promote tumour progression besides anti-tumoural M1 subsets. Selective depletion or reprogramming of M2 may represent an innovative anti-cancer strategy. The actin cytoskeleton is central for cellular homeostasis and is targeted for anti-cancer chemotherapy. Here, we show that targeting G-actin nucleation using chondramide A (ChA) predominantly depletes human M2 while promoting the tumour-suppressive M1 phenotype. ChA reduced the viability of M2, with minor effects on M1, but increased tumour necrosis factor (TNF)α release from M1. Interestingly, ChA caused rapid disruption of dynamic F-actin filaments and polymerization of G-actin, followed by reduction of cell size, binucleation and cell division, without cellular collapse. In M1, but not in M2, ChA caused marked activation of SAPK/JNK and NFκB, with slight or no effects on Akt, STAT-1/-3, ERK-1/2, and p38 MAPK, seemingly accounting for the better survival of M1 and TNFα secretion. In a microfluidically-supported human tumour biochip model, circulating ChA-treated M1 markedly reduced tumour cell viability through enhanced release of TNFα. Together, ChA may cause an anti-tumoural microenvironment by depletion of M2 and activation of M1, suggesting induction of G-actin nucleation as potential strategy to target tumour-associated macrophages in addition to neoplastic cells.

Tumours are composed of proliferating neoplastic cells dynamically interacting with stromal cells^1^, which promote cancer progression by creating tumourigenic microenvironments^2^,^3^. Tumour-associated macrophages (TAM) represent a key component of tumour-supporting stroma^4^ and derive from monocyte recruitment, infiltration and differentiation by tumour-produced chemotactic and differentiation agents such as monocyte chemotactic protein-1 (MCP-1) and macrophage colony-stimulating factor (M-CSF), respectively. Macrophages may display both pro-tumoural and anti-tumoural effects. For simplicity, the phenotypic heterogeneity is described as gradient between two opposite polarizations defined as M1 and M2. The M1 subset is obtained by stimulation with Toll-like receptor (TLR) ligands and interferon (IFN)γ, produces immune-stimulating cytokines, and exhibits anti-tumoural activity by supporting anti-tumour immunity. M2 are instead induced by Th2-type cytokines (e.g., interleukin (IL)-4) and exert immune-suppressive and pro-tumoural effects^4^. The tumour milieu causes conditioning of infiltrating macrophages into an immunosuppressive M2 phenotype, and macrophage infiltration often correlates with poor cancer prognosis^5^,^6^. Thus, macrophages are currently considered as promising target for chemotherapy, aiming to reduce M2 at tumour sites and to enhance M1 activity^4^,^7^,^8^. Ideally, an anti-tumour therapy should suppress viability and/or limit invasion of cancer cells but also adjust macrophage functions at once.

The actin cytoskeleton is central for cellular homeostasis and represents a point of intervention for chemotherapy with cancer cells^9^. However, actin dynamics involving monomeric actin (G-actin), actin nucleation, and polymerization into filaments (F-actin) are significant events in different cells and biological processes (e.g., cytokinesis, motility, endocytosis)^10^. Given the key role of actin in cellular functions and the requirement of migration, differentiation and polarization to establish tumour-promoting activities of macrophages, we here hypothesized that a chemotherapeutic approach targeting actin may simultaneously affect cancer cell and macrophage biology. To test this hypothesis, we utilized chondramide A as tool (ChA; Suppl. Fig. 1), an actin-targeting compound with unique actin-perturbing properties^11^,^12^, and we evaluated its effects on functions of human macrophages. Chondramides are cell-permeable cyclic depsipeptides from *Chondromyces*, assumed to act by F-actin stabilization^11^,^13^ and being cytotoxic for tumour cells^13^,^14^,^15^. Recently, we showed that ChA at a low dose of 0.75 mg/kg (given i.p. three times a week) efficiently reduced tumour growth and decreased tumour angiogenesis *in vivo*^16^,^17^,^18^, supporting the potential of ChA as cancer therapeutics.

Here, we demonstrate that ChA i) blocks monocyte chemotaxis, ii) kills M2, iii) activates nuclear factor kappa (NFκ)B and stress-activated protein kinases (SAPK)/c-Jun N-terminal kinase (JNK) in M1, but not in M2, connected with TNFα secretion, and iv) increases M1-mediated cytotoxicity for cancer cells in a TNFα-dependent fashion. These unique properties of ChA might be connected to direct binding to G-actin and induction of G-actin nucleation.

## Results

### ChA suppresses tumour cell viability and monocyte migration

**Figure 1:**
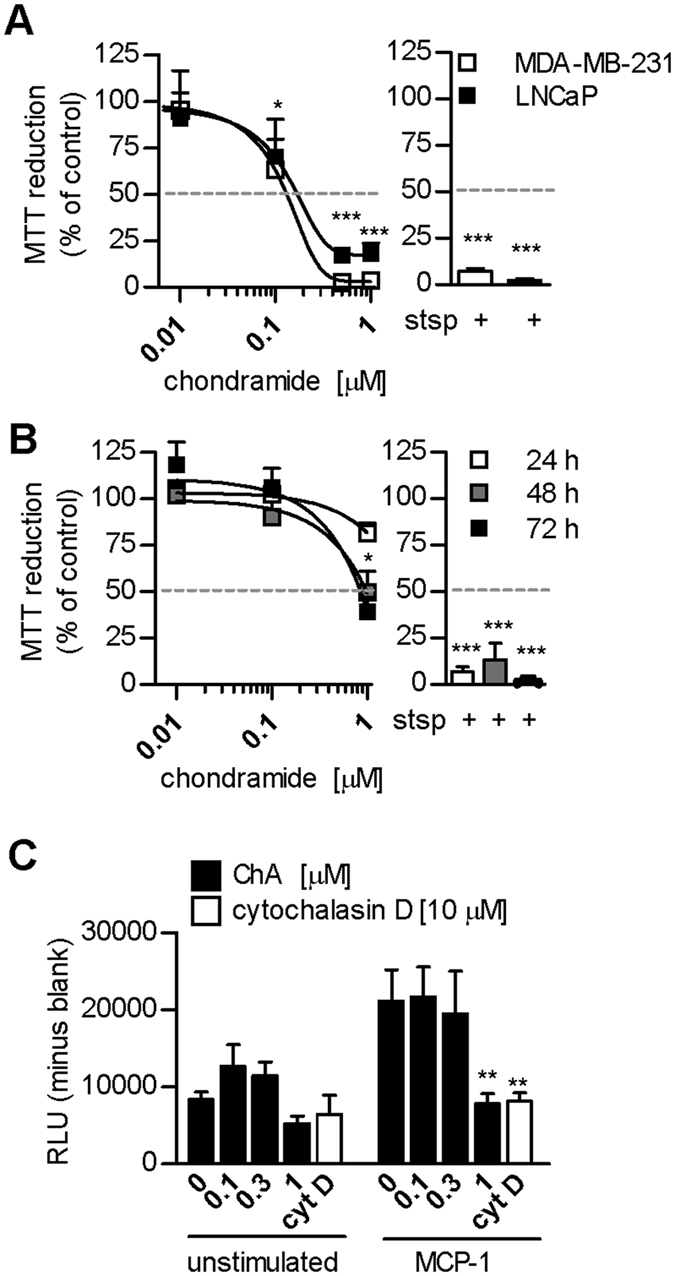
ChA induces cancer cell death and suppresses monocyte migration. (**A**,**B**) MTT assay after incubation with ChA at the indicated concentrations or staurosporine (stsp, 3 μM) of (**A**) MDA-MB-231 and LNCaP for 48 h or of (**B**) human monocytes, for the indicated periods (24, 48 or 72 h). (**C**) Monocyte migration in Boyden chambers. Monocytes (upper chamber) were pre-incubated with test compounds or vehicle (0.1% DMSO) and exposed to MCP-1 (lower chamber) or left untreated for 2 h. Then, the number of migrated monocytes in the lower chamber was evaluated by ATP determination using CellTiter-Glo Luminescent Cell Viability Assay. Data are expressed as percentage of vehicle control (**A**,**B**) or as relative light units (RLU) (**C**), means + SEM; n = 3; *p < 0.05, **p < 0.01, ***p < 0.001 vs vehicle control.

Dual targeting of TAMs and cancer cells is a promising therapeutic approach for solid tumours that are infiltrated by macrophages^19^,^20^,^21^. The viability of the cancer cell lines MDA-MB-231 and LnCAP was reduced by ChA with EC_50_ ≈ 0.2 μM (Fig. 1A). Interestingly, monocytes were less sensitive and partial loss of viability was only observed at 1 μM ChA after 48 or 72 hrs (Fig. 1B). We then analyzed monocyte migration, the initial event for macrophage infiltration. Upon MCP-1 exposure, monocyte migration was increased about 3-fold, and this was completely blocked by 1 μM ChA (Fig. 1C). Note that 1 μM ChA did not significantly impair monocyte viability within 24 hrs, excluding cytotoxicity as reason for reduced monocyte migration.

### ChA reduces viability of M2 but hardly of M1

**Figure 2:**
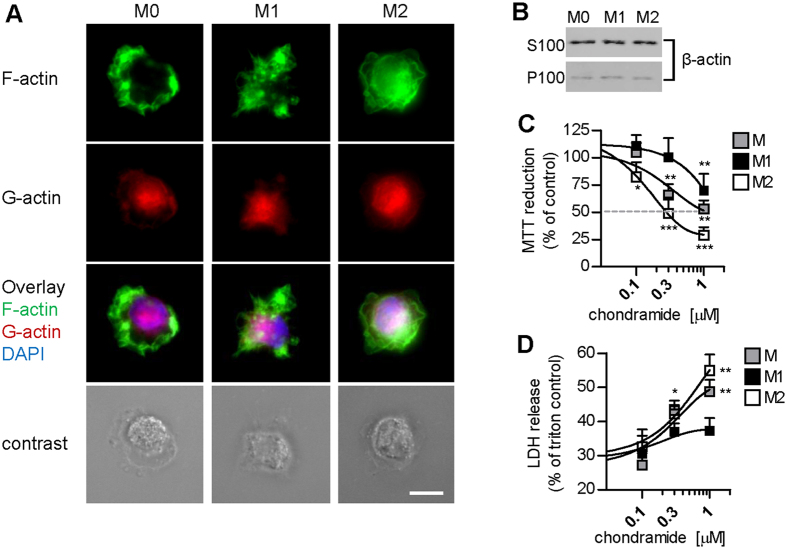
ChA induces lytic cell death of M2 but hardly affects M1 viability. (**A**) F- and G-actin localization in polarized M0, M1 and M2. The staining was performed after cell fixation with Alexa Fluor 488 phalloidin (green, F-actin), Alexa Fluor 594 DNase I (red, G-actin), and DAPI (blue, nuclei); n = 3; scale bar, 10 μm. (**B**) Analysis of soluble (S100) and insoluble (P100) actin pools in macrophages after cell lysis, centrifugation (100,000 × g) and Western blot. Pictures are representative of three independent experiments. (**C**) MTT and (**D**) LDH assay of M0, M1 and M2 after incubation with ChA for 48 h (MTT) or 24 h (LDH). ChA was added 30 min prior to polarization. Data are expressed as percentage of (**C**) vehicle (0.1% DMSO) or (**D**) Triton X-100 (1%) controls, means + SEM; n = 4; *p < 0.05, **p < 0.01, ***p < 0.001 vs vehicle control.

Next, we assessed macrophage viability in relation to actin cytoskeleton arrangement. Monocytes were differentiated and were left unpolarized (M0) or polarized to M1 and M2 by incubation with LPS/IFNγ or IL4, respectively. M1 presented both filopodia and lamellipodia, and F-actin was distributed through the cytoplasm, while M2 were round in shape and similar to M0, with perinuclear F-actin and at the lamellar processes (Fig. 2A). G-actin was instead present in the nuclear and perinuclear region, with a comparable distribution pattern regardless of the polarization state. Notably, the total amounts of soluble and insoluble cellular actin among macrophage phenotypes were comparable (Fig. 2B).

ChA strongly reduced the viability of M2 (EC_50_ = 0.2 μM, Fig. 2C). Interestingly, M0 were less susceptible to ChA (EC_50_ ~ 1 μM), and M1 were hardly influenced (EC_50_ > 1 μM, Fig. 2C). These effects of ChA were accompanied by a major loss of membrane integrity in M0 and M2, but hardly in M1, observed by both analysis of LDH release (Fig. 2D) and cell morphology (Suppl. Fig. 2). Of interest, M1 morphology was profoundly altered by ChA and presented surface blebbing, but these cells remained essentially vital (Fig. 2D, Suppl. Fig. 2). Thus, ChA at 0.1 μM acts as cytotoxic agent for M2, but hardly for M1 even at higher concentrations (1 μM). Similar features as for M2 were observed for M0 that, after differentiation with M-CSF, may behave like M2^22^,^23^,^24^.

**Figure 3:**
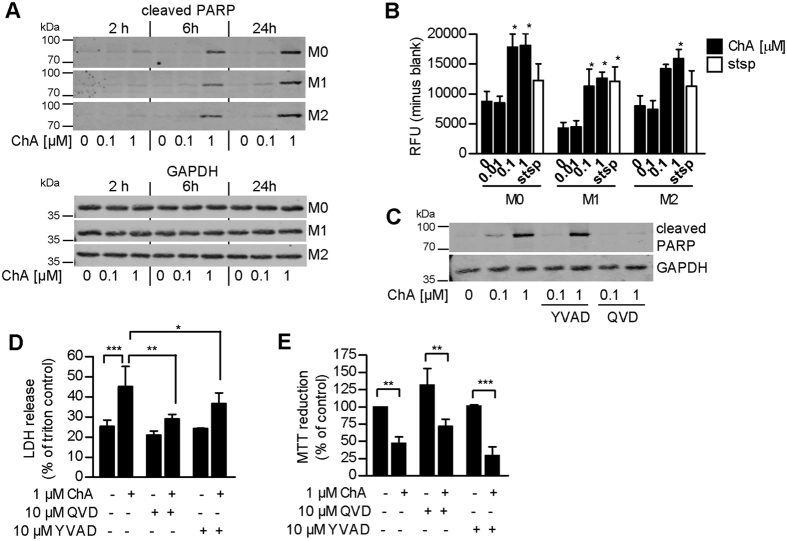
ChA activates effector caspases but induces caspase-independent cell death of M2. (**A**) PARP cleavage and (**B**) caspase-3/7 activation in macrophages after treatment with vehicle (0.1% DMSO) or ChA for (**A**) the indicated times or (**B**) 24 h. ChA or staurosporine (stsp) were added 30 min prior to polarization. Pictures shown in (**A**) are representative Western blots for cleaved PARP and GAPDH (for normalization) of three independent experiments. In (**B**), caspase-3/7 activation was determined by using Apo-ONE Homogenous Caspase-3/7 assay; data are means + SEM; n = 3. (**C**) PARP cleavage, (**D**) LDH and (**E**) MTT assays of M2 pre-treated (30 min, 37 °C) with vehicle (0.1% DMSO), the pan-caspase inhibitor QVD, or the caspase-1 inhibitor YVAD (10 μM, each), and incubated with vehicle (0.1% DMSO) or ChA for (**C**,**D**) 24 h or (**E**) 48 h. ChA was added 30 min prior to polarization or corresponding incubations without stimuli (for M0). Western blots for cleaved PARP or GAPDH shown in (**C**) are representative of three independent experiments. In (**D**,**E**) data are expressed as percentage of (**D**) Triton X-100 (1%) or (**E**) vehicle controls, means + SEM; n = 3; *p < 0.05, **p < 0.01, ***p < 0.001 vs vehicle control.

Interestingly, ChA induced PARP cleavage after 6 and 24 hours in all macrophage subsets (Fig. 3A), accompanied by activation of caspase-3/7 (Fig. 3B). These activities were blocked by the pan-caspase inhibitor QVD, while only minor or no effects were observed by the caspase-1 inhibitor YVAD (Fig. 3C). QVD, and partially also YVAD, significantly reduced ChA-mediated membrane damage in M2 (LDH release, Fig. 3D), but did not restore cell viability (MTT assay); ChA lowered viability also when caspases were inhibited (Fig. 3E).

### ChA induces G-actin nucleation and disruption of dynamic F-actin structures in macrophages

**Figure 4:**
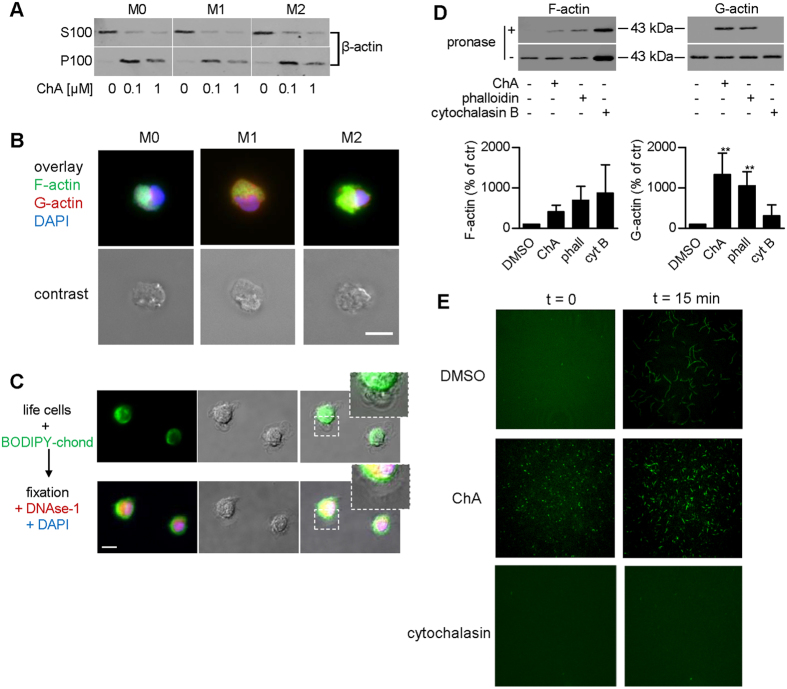
ChA reduces soluble G-actin pools and disrupts dynamic F-actin structures on macrophage surfaces. (**A**) Analysis of soluble (S100) and insoluble (P100) actin pools. Cells were pre-treated (30 min) with vehicle (0.1% DMSO) or ChA at the indicated concentrations prior to polarization. After 24 h, proteins in M0, M1 or M2 were analyzed by Western blot. (**B**) F- and G-actin localization in M0, M1 or M2 after pre-treatment (30 min) with vehicle (0.1% DMSO) or 0.1 μM ChA prior to polarization. After 24 h, cells were fixed and staining was performed (F-actin, green; G-actin, red; nuclei, blue); scale bar, 10 μm. Pictures shown are representative of three independent experiments. (**C**) Live cell imaging of macrophages (M0) with a cell-permeable BODIPY-modified ChA analogue (1 μM, green, incubated for 20 min), followed by fixation and G-actin (red) and DNA staining (blue); n = 3; scale bar, 10 μm. (**D**) DARTS of G- and F-actin in presence of vehicle (0.1% DMSO), 1 μM ChA, 10 μM phalloidin or 1 μM cytochalasin B. For densitometry analysis (lower panels), bands were normalized to signals of matching undigested controls; data, means + SEM, n = 3; **p < 0.01 vs vehicle control. (**E**) ChA (5 μM) enhances actin nucleation in a cell-free assay from t = 0.5 (left panels) within 15 min (right panels), while cytochalasin D (1 μM) blocks it. Pictures shown are representative of three independent experiments.

The differential cytotoxic effects of ChA in M1 and M2 let us to further study its interference with actin. ChA caused loss of the amounts of soluble but increase of insoluble actin (Fig. 4A), suggesting an accumulation of F-actin at the expense of G-actin. However, dynamic F-actin structures on the surface of macrophages were disrupted by ChA (Fig. 4B), which was paradoxical as compared to the view of chondramides being F-actin stabilizers. Instead, ChA caused accumulation of F-actin lumps in the nuclear and perinuclear region, where G-actin was localized, which was more pronounced for M2 and M0 as compared to M1 (Fig. 4B).

Next, we performed live cell imaging using a BODIPY-modified ChA analogue as probe (Suppl. Fig. 3A)^25^. The ChA probe mainly localized in the perinuclear region, in close proximity to G-actin pools (Fig. 4C), suggesting interference with G-actin. The staining of the fluorescent ChA probe was abolished after addition of non-fluorescent ChA, indicating common binding sites (Suppl. Fig. 3B). F-actin structures on the cell surface were not stained by fluorescent BODIPY-ChA in living macrophages, though such staining was obtained when the probe was added after fixation of the cells, suggesting slow F-actin on-rate of ChA (Suppl. Fig. 3C).

To confirm ChA binding to G-actin, we used a DARTS approach^26^, which validates ligand-target interaction by decreased susceptibility of target protein(s) against protease digestion due to ligand-binding. Thus, separated G- and F-actin pools were incubated with ChA, phalloidin or cytochalasin B and treated with pronase. Both ChA and phalloidin strongly protected G-actin from proteolytic degradation, but had only minor effects on F-actin (Fig. 4D), while cytochalasin B solely protected F-actin. Cell-free TIRF microscopy analysis revealed that in untreated samples, G-actin nucleation is almost absent at the beginning of the experiment, but after 15 min, actin filaments are formed (Fig. 4E). However, after incubation with 5 μM ChA, actin nuclei were rapidly (after 30 sec) apparent and the length of actin filaments after 15 min was considerably smaller as compared to untreated controls (Fig. 4E), seemingly due to actin consumption by heavy nucleation. In contrast, cytochalasin D, used as control, blocked inhibition of nucleation and polymerization (Fig. 4E), as expected. Together, ChA may primarily target G-actin and induce its nucleation/polymerization.

Finally, ChA caused cell contraction and loss of lamellar processes in living cells already 10–30 min after exposure (Suppl. Fig. 4A). Interestingly, ChA induced the formation of an open perinuclear actin ring (Suppl. Fig. 4B), nuclear division and cellular separation (Suppl. Fig. 4A,B) in macrophages independent of their polarization state. Note that cellular collapse was not evident, despite a profoundly altered morphology as compared to control cells and cytochalasin B-treated macrophages (Suppl. Fig. 4C).

### ChA increases the amount of TNFα released by M1

**Figure 5:**
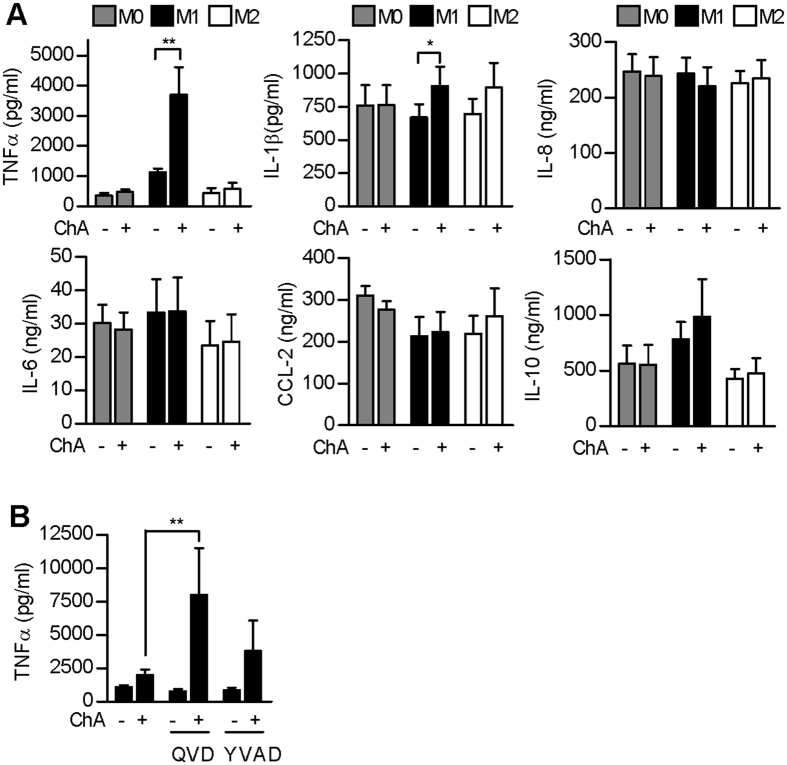
ChA stimulates M1 for TNFα release. (**A**) Macrophages (M0) were pre-treated with vehicle (0.1% DMSO) or 1 μM ChA for 30 min prior to polarization. After 24 h, the levels of TNFα, IL-1β, IL-8, IL-6, MCP-1 (CCL-2), and IL-10 in cell culture supernatants of M0, M1 and M2 were determined by ELISA. Data are means + SEM; n = 4; *p < 0.05, **p < 0.01; ANOVA + Bonferroni. (**B**) TNFα release from M1 pre-treated (30 min) with vehicle, QVD or YVAD (10 μM, each), followed by addition of 1 μM ChA for 30 min and 24 h M1 polarization. Data, means + SEM; n = 4; **p < 0.01.

Because TAMs influence the tumour environment by releasing growth factors, chemokines and cytokines, we next analyzed whether ChA affects the secretion of tumour-relevant cytokines from macrophages. ChA significantly elevated TNFα release by M1, but hardly in M0 and M2 (Fig. 5A). ChA also slightly increased IL-1β in M1, and to some extent also in M2. In contrast, IL-8, IL-6, MCP-1, and IL-10 were essentially unaffected, regardless of macrophage polarization. Note that the increased TNFα release from M1 was not related to cytotoxicity due to membrane rupture (Fig. 2D) and the caspase inhibitor QVD (and partially also YVAD) rather increased ChA-induced TNFα secretion (Fig. 5B). Moreover, ChA failed to enhance ROS formation, excluding induction of general oxidative stress (Suppl. Fig. 5).

We next evaluated whether other anti-tumoural or actin-interfering agents display a similar profile of macrophage toxicity and TNFα release. Staurosporine and gliotoxin caused death of macrophages independent of the polarization state (Suppl. Fig. 6A,B) and suppressed the release of cytokines in M1 (Suppl. Fig. 6C). Interestingly, cytochalasin B that inhibits polymerization/depolymerization of F-actin, and latrunculin B, which prevents F-actin assembly, also reduced cell viability selectively of M2 comparable to ChA (Suppl. Fig. 6D). However, cytochalasin B and latrunculin B caused loss of membrane integrity in all macrophage phenotypes independent of the polarization state (Suppl. Fig. 6E), and also failed in enhancing TNFα release in M1 (Suppl. Fig. 6F).

### ChA elevates the activation of select signaling pathways relevant for survival and TNFα secretion in M1 but not in M2

**Figure 6:**
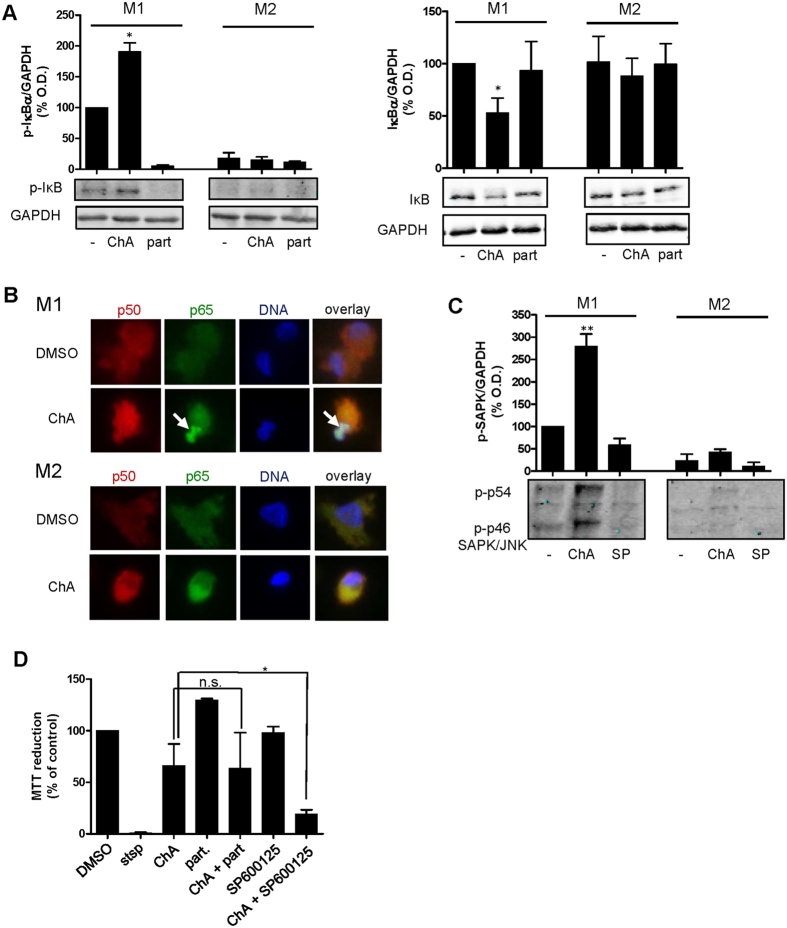
ChA causes activation of NFκB and SAPK/JNK in M1. (**A**) M0 were pre-treated with 1 μM ChA, 10 μM parthenolide (part), or vehicle (0.5% DMSO) for 30 min and polarized to M1 or M2. After 30 min, the amounts of phosphorylated IκB (left panel) and after 3 h the total protein levels of IκB (right panel) were assessed by Western blot. Densitometric data (normalized to GAPDH) are expressed as percentage of vehicle control; means + SEM; n = 3. (**B**) Determination of p50 (red), p65 (green) and nuclear DNA (blue) after 30 min incubation with 1 μM ChA, 10 μM parthenolide (part) or 0.5% DMSO (vehicle) in M1 and M2. Results are representatives for three independent experiments. Arrows indicate accumulated p65 in the nucleus. (**C**) M0 were pre-treated with 1 μM ChA, 10 μM SP600125 or 0.5% DMSO for 30 min, polarized to M1 or M2, and after 30 min analyzed for phosphorylated SAPK/JNK by Western blot. Densitometric data for phosphorylated SAPK/JNK (normalized to GAPDH) are expressed as percentage of vehicle control; means + SEM; n = 3. (**D**) MTT assay for M1 pre-treated with 1 μM ChA, 10 μM parthenolide, 10 μM SP600125 or vehicle for 30 min, and polarized to M1 for 48 h. Data are expressed as percentage of vehicle control (0.1% DMSO); means + SEM; n = 3. *p < 0.05; **p < 0.01.

Because M1 were resistant against cell death induction, implying that survival signaling pathways might be operative, we assessed the activation status of signaling molecules that are critically involved in cell survival and pro-inflammatory functionalities (e.g., TNFα release). In M1, but not in M2, ChA enhanced the phosphorylation of IκBα accompanied by consequent decreased protein levels of IκBα (Fig. 6A), and caused nuclear enrichment of the p65 subunit of NFκB (Fig. 6B). Similarly, ChA induced significant phosphorylation of SAPK/JNK in M1 with only minor effects in M2 (Fig. 6C). Also STAT-3 phosphorylation was increased by ChA in M1 but not in M2, whereas Akt phosphorylation was slightly elevated in both phenotypes (Suppl. Fig. 7A,B). ERK-1/2 and STAT-1 phosphorylation was not affected by ChA, regardless of macrophage polarization, and p38 MAPK phosphorylation was increased by ChA in M2 (Suppl. Fig. 7C–E).

The enhanced signaling of NFκB and SAPK/JNK may explain the better survival of M1 after ChA treatment. Therefore, we blocked these survival pathways using respective inhibitors and assessed cell viability after ChA treatment. Inhibition of SAPK/JNK by SP600125 in M1 facilitated cell death induction by ChA, while inhibition of the NFκB pathway by parthenolide was without effect (Fig. 6D).

### ChA causes activation of M1 cells and enhances M1-mediated cytotoxicity against MCF-7 cells in a microfluidically biochip assay

**Figure 7:**
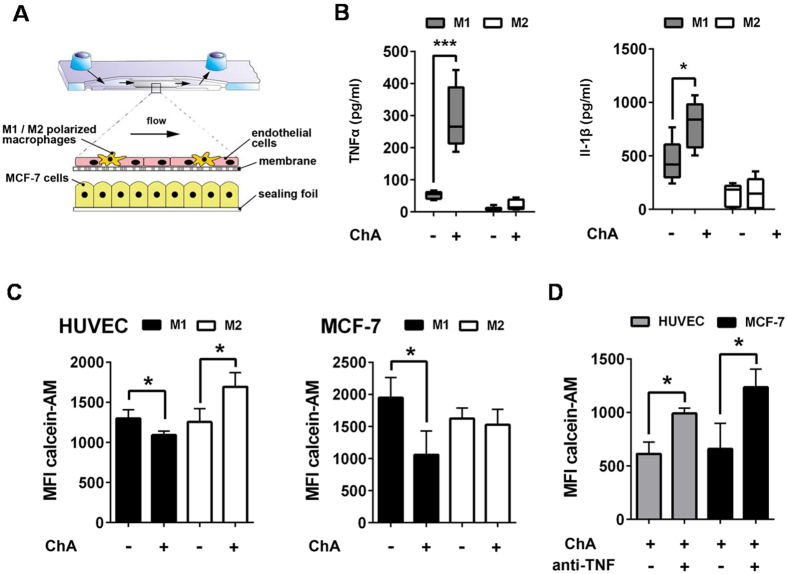
ChA triggers TNFα release by M1 and reduces MCF-7 viability in a biochip-based tumour model. (**A**) Schematic view of the tumour biochip model. The endothelial cell layer is formed by HUVEC and polarized macrophages and co-cultured on a suspended membrane within a microfluidically supported biochip. MCF-7 cells are cultured underneath the vascular layer at the bonding foil of the biochip. (**B**) Release of TNFα and IL-1β upon treatment with ChA (1 μM) or vehicle (0.1% DMSO) of tumour models composed of M1 or M2 and MCF-7 cells after 48 h. TNFα and IL-1β in the MCF-7 compartment were analyzed by CBA. (**C**,**D**) Viability of MCF-7 cells and HUVEC by Calcein-AM staining after 48 h incubation with M1 or M2 in the presence 1 μM ChA or vehicle (0.1% DMSO). Mean fluorescence intensity (MFI) was measured by random field analysis of 30 regions of interest per experiment. Data, means + SEM, n = 3. (**D**) Effects of a TNFα-neutralizing antibody on the viability of HUVEC and MCF-7 cells of tumour models (Calcein-AM staining) after 48 h containing M1 upon ChA (1 μM) treatment. Data, means + SEM; n = 3. *p < 0.05.

In order to evaluate how ChA treatment of macrophages influences cancer cells in co-cultures, we utilized a microfluidically supported biochip assay^27^ and designed it as a dynamically perfused three-dimensional human tumour model. Macrophages were co-cultured with HUVEC and polarized towards M1 or M2. MCF-7 cells, resembling the tumour component, were cultured underneath the HUVEC-macrophage cell layer (Fig. 7A).

ChA (1 μM) induced release of TNFα and IL-1β in models containing M1 that was not observed with M2 (Fig. 7B). No significant effects of ChA were found for the release of IL-6 or IL-10 (Suppl. Fig. 8A). We further analyzed the functional consequences of TNFα and IL-1β release on cell viability using Calcein-AM staining. ChA significantly reduced viability of HUVEC and MCF-7 cells in models containing M1 (Fig. 7C, Suppl. Fig. 8B,C). Moreover, ChA treatment in the presence of M1 was associated with diminished expression of the adherens junction protein VE-cadherin, indicating a loss of vascular barrier function and increased permeability (Suppl Fig. 8B). In contrast, in models containing M2, the viability of HUVEC was rather enhanced by ChA and no impact on the viability of MCF-7 cells was observed. Moreover, also when heterogeneous M1/M2 populations were applied, ChA treatment significantly reduced MCF-7 cell viability (Suppl. Fig. 8E) accompanied by increased TNFα levels. Note that in the absence of macrophages, ChA treatment did not affect MCF-7 cell viability (Suppl. Fig. 8D).

The pronounced TNFα release by ChA-treated M1 and the fact that TNFα mediates apoptosis and tumour cytotoxicity prompted to investigate whether TNFα might contribute to the decreased viability of MCF-7 cells in the ChA-treated M1 model. In fact, depletion of TNFα by neutralizing antibodies prevented loss of MCF-7 cell viability upon ChA-treatment (Fig. 7D).

## Discussion

TAMs as a major infiltrate in solid tumours and the concept of macrophages contributing to tumour progression has been integrated in cancer therapy^28^. Despite the potential of this approach, there is currently an unmet need for identification of targets for intervention with macrophages. Several efforts are made to reveal basic molecular and cellular mechanisms allowing selective interference with M1 and M2 responses. Recent data have shown the possible involvement of Notch^29^ and mammalian target of rapamycin (mTOR)^30^. Currently, integrative strategies targeting monocytes/macrophages in cancer therapy essentially rely on previous studies in the inflammation field and aim to suppress the infiltration of monocytes into the tumour by targeting tumour-promoting inflammation (e.g., by anti-inflammatory drugs)^31^. Other clinical-relevant alternatives aim at depleting macrophages by toxic effects on monocytes by the natural product trabectedin^32^ or on macrophages by the bisphosphonate zoledronic acid^33^, which however, do not allow to discriminate between cellular subtypes. As opposite approach, attempts have been made to stimulate macrophages for higher TNFα production, for example by TLR-9 activators and CpG oligodeoxynucleotides^34^,^35^, aiming to promote immune reactions against the tumour.

Our data suggest that induction of G-actin nucleation might represent a possible approach for simultaneously targeting relevant events in different macrophage subtypes (i.e., reduction of macrophage infiltration by suppression of monocyte migration, killing of M2, and enhancement of TNFα release by M1) and thereby inducing cancer cell death. In fact, using a microfluidically supported biochip designed as dynamically perfused three-dimensional tumour model, we show that ChA treatment of M1, but not of M2, causes marked TNFα secretion and loss of viability of MFC-7 cells, an effect that could be reversed by selective TNFα neutralization using TNFα antibodies.

As compared to other actin-targeting agents, chondramides are cell-permeable (cfr. phalloidins), can be reasonably produced by fermentation (cfr. jasplakinolide^36^) and their biosynthetic genes in *Chondromyces crocatus* have been cloned^15^. Chondramides have been chemically characterized and synthesized^11^,^12^,^36^, and are cytotoxic for cancer cells^12^,^13^,^14^,^15^,^16^,^17^,^18^. Moreover, ChA showed efficacy *in vivo*, as it reduced the growth and vascularization of tumors in mice^16^,^17^,^18^. Therefore, ChA allows to exploring the possibility to attack the actin cytoskeleton as an approach to resolve the cellular and molecular heterogeneity of macrophages and cancer cells. In fact, actin is a key player of cellular shape and movement, which are cellular processes required for macrophage infiltration in tumours and for invasive tumour migration of cancer cells^9^. Importantly, the observed cellular effects appeared, however, not simply related to actin targeting, instead they were dependent of the specific mechanism of ChA/actin interference.

Chondramides are assumed to act by binding and stabilization of F-actin^11^,^13^. Computational docking indicated a binding site for chondramide C (Suppl. Fig. 1) similar to phalloidin, namely in the cavity formed by three actin proteins in the filament^11^. Here, we observed a disruption of dynamic F-actin structures on the macrophage surface, which appeared not promptly explained by the F-actin stabilizing action of ChA. DARTS experiments indicated G-actin as binding partner for ChA and microscopic analysis using a chondramide-fluorescent probe suggested the perinuclear region (where both G-actin and static F-actin are present) as possible localization area in macrophages. Moreover, cellular treatment with ChA caused a prominent loss of soluble G-actin, and TIRF microscopy indicate rapid and massive induction of G-actin nucleation by ChA. Together, in macrophages ChA may primarily act by depleting the pool of available G-actin monomers through induction of G-actin nucleation and/or hyper-polymerization of static perinuclear F-actin. Actin nucleation involves three G-actin monomers^9^, and is thus compatible with the suggested binding site of chondramides^11^. Accordingly, actin nucleation was previously observed for phalloidin^37^ and jasplakinolide^38^, which seemingly share the actin-trimer binding. In this view, disruption of F-actin on the macrophage surface by ChA would be due to insufficient amounts of G-actin to guarantee a balanced turnover of dynamic actin filaments.

ChA also altered the actin cytoskeleton in potoroo kidney epithelial cells^13^ and A-498 cancer cells^12^. In these cell types, however, the major changes were mainly regarded as loss of stress fibers followed by formation of actin aggresomes and were observed only after hours of treatment. For macrophages, instead, the effects of ChA were extremely rapid (10–30 min), probably reflecting the fast turnover of actin filaments which is required for macrophage dynamics (e.g., movement, phagocytosis). Note that mouse bone-marrow-derived M2 possess a higher intrinsic motility than M1, seemingly related to different cytoskeletal functions^39^. In our experiments, however, similar early events in M1 and M2 after ChA treatment were observed, i.e. reduction of the cell size and formation of a rather organized perinuclear actin ring. Both the weakening of plasma membrane/cortical actin interactions and the actin ring may then produce the driving force for budding and cellular division. Interestingly, these events partially resemble morphological hallmarks of apoptosis, but were preceding PARP cleavage and caspase activation and, despite major cell changes, they were not necessarily associated with cytotoxicity (e.g., in M1). This highlights the actin cytoskeleton as mediator of morphological changes accompanied by cell death, and not only as apoptosis sensor^40^.

Despite similar early events in macrophage subsets, different susceptibilities were observed in terms of ChA-induced cytotoxicity. Although caspase-dependent PARP cleavage was observed in all these phenotypes, microscopic analysis and LDH release essentially indicated that M0 and M2 underwent a lytic necrotic event. In contrast, M1 were hardly affected and presented only some blebbing at later time points, which may be due to either minor cell damage (as suggested by slightly lower F-actin lumps) and/or the induction of survival mechanism along with LPS/IFNγ. In fact, in M1, ChA increased the activation of the survival pathways NFκB and SAPKK/JNK, and to a lower extent also Akt and STAT-3. Increased NFκB activation may account also for the higher TNFα production in M1. Note that none of the signaling molecules were markedly affected by ChA in M2, and other major signaling routes including p38 MAPK, ERK-1/2 or STAT-1 were not influenced in M1. Taken together, our findings suggest G-actin-targeting as a novel possible mechanism for selective killing of M2 and positive regulation of M1 activities that might be connected to cytotoxicity for cancer cells, with potential relevance for tumour immunology.

## Materials and Methods

### Cells

Human umbilical vein endothelial cells (HUVEC) were isolated from human umbilical cords and cultured for up to four passages as described previously^27^. Donors were informed about the aim of the study and gave written consent. Human monocytes were isolated from peripheral blood obtained from healthy volunteers. The protocols for experiments with HUVEC and monocytes were approved by the ethical commission of the Friedrich-Schiller-University Jena. All methods were performed in accordance with the relevant guidelines and regulations. Leukocytes were immediately concentrated by centrifugation (4,000 × g/20 min/20 °C) of freshly withdrawn blood. Peripheral blood mononuclear cells (PBMC) were then isolated by dextran sedimentation and centrifugation on lymphocyte separation medium (PAA Laboratories, Pasching, Austria), and monocytes were separated by adherence to culture flasks, which gave a purity of >85%, defined by forward- and side-light scatter properties and detection of the CD14 by flow cytometry^41^. Macrophages were obtained by culturing monocytes in RPMI 1640 (Sigma-Aldrich, Taufkirchen, Germany) containing 5% human serum (Sigma-Aldrich), 2 mM L-glutamine, 100 μg/ml penicillin/streptomycin (PAA Laboratories) supplemented with 25 ng/ml M-CSF (Pepro Tech, Hamburg, Germany) for 6 days. Macrophage polarization was obtained by addition of 100 ng/ml lipopolysaccharide (LPS; from Escherichia coli 0127:B8; Sigma-Aldrich) plus 100 ng/ml interferon-γ (IFN-γ; Pepro Tech) for M1 or with 20 ng/ml interleukin-4 (IL-4; Pepro Tech) for M2 polarization^42^. To assure correct polarization, cells were assessed by flow cytometry for expression of the M2 surface markers CD163 and CD206, while M1 were characterized by high expression of CD54 and CD80^43^.

MDA-MB-231, MCF-7, and LNCaP cells were obtained at LGC Standards (Wesel, Germany). Authentication of cell lines was carried out by STR analyses (LGC Standards) and experiments were completed within 4 months of receipt of cell lines. Cells were cultured in RPMI 1640 (MDA-MMB-231 and LNCaP) or DMEM-HG (MCF-7; Gibco, Waltham, MA) supplemented with 10% fetal calf serum (FCS), 2 mM L-glutamine, 100 μg/ml penicillin/streptomycin, 10 mM HEPES, and 1 mM sodium pyruvate at 37 °C in a humidified atmosphere and 5% CO_2_ in air.

### MTT assay

Monocytes (10^5^ cells/100 μl) were seeded in 96-well plates and differentiated towards macrophages. LNCaP (2 × 10^5^ cells/ml) and MDA-MB-231 (5 × 10^3^ cells/100 μl) were seeded in 96-well plates. Cells were treated with vehicle (0.05% DMSO) or ChA at 37 °C and analyzed as reported in Supporting Information.

### Lactate dehydrogenase release (LDH) assay

After treatment of macrophages, cell culture supernatants were centrifuged (600 × g, 5 min, 4 °C), and 100 μl of supernatant were mixed with 200 μl LDH reaction buffer (75 mM Tris/HCl, pH 7.4, 0.3 mM NADH, 1.5 mM Na-pyruvate) in a 96-well plate. The decrease of NADH was measured immediately by absorbance at 340 nm in a kinetic mode (every 7 s for 20 min; Multiskan Spectrum).

### Monocyte migration assay

Monocyte migration in response to MCP-1 (100 ng/ml, PeproTech) was evaluated using the microplate system ChemoTx^®^ (Neuro Probe, Inc., Gaithersburg, MD), where microchambers were separated from the upper compartment by polycarbonate membranes (pore size 5 μm) as described^44^. For more details, see Supporting Information.

### Fluorescence microscopy

Macrophages were adhered onto Petri dishes with glass bottom or glass coverslips in a 12-well plate, pre-incubated with vehicle (0.1% DMSO) or test compounds, incubated with BODIPY-chondramide, and life-cell imaging was performed. Alternatively, cells were fixed in 4% formaldehyde, permeabilized with 0.2% Triton X-100, and stained with Alexa Fluor 488 phalloidin and Alexa Fluor 594 DNase I conjugates. DNA was stained with 0.6 μg/ml DAPI. Fluorescence was visualized with a Zeiss Axio Observer. Z1 microscope and a LCI Plan-Neofluar 63×/1.3 Imm Corr DIC M27 Objective (Carl Zeiss AG, Jena, Germany). For more details, see Supporting Information.

### Microfluidically supported biochip assay

Biochips were made by injection moulding from cyclo olefin polymer (COP) Zeonor^®^, obtained from microfluidic ChipShop GmbH (Jena, Germany), and manufactured as described previously^27^. HUVEC and monocytes were mixed and seeded at top of the biochip embedded membrane. Subsequently MCF-7 cells were seeded at the lower bonding foil of the biochip. After differentiation of macrophages in presence of M-CSF, M1 polarization was induced by addition of IFNγ and M2 polarization was induced by addition of IL-4. Medium containing 1 μM ChA or vehicle was perfused over the vascular layer with a shear stress rate of 3 dyn/cm^2^ for 2 h. Supernatants were collected after indicated times for cytokine measurement using colorimetric bead assay. Cell layers were analyzed by immunofluorescence microscopy for VE-cadherin expression and calcein-AM staining. For details, see Supporting Information.

### Western Blot

Macrophages were lysed with 1% NP-40 and centrifuged (10,000 × *g*, 5 min, 4 °C). The supernatant was mixed with 4 × SDS-PAGE sample loading buffer, heated to 95 °C for 5 min, and analyzed by SDS-PAGE on a 10% gel followed by protein transfer onto nitrocellulose membrane. Antibodies: GAPDH (#sc-365062, Santa Cruz Biotechnology, Heidelberg, Germany), β-actin (#4967 S), inhibitor of kappa B α (IκBα, #4814 S), phosphorylated (phospho-)IκBα (Ser32/Ser36, #2859 S), phosphorylated signal transducer and activator of transcription (STAT)-1 (Tyr701, #9171), phosphorylated STAT-3 (Tyr705, #9145 S), phosphorylated p38 mitogen-activated protein kinase (MAPK) (Thr180/Tyr182, #9211), phosphorylated extracellular signal-regulated kinase (ERK)-1/2 (Thr202/Tyr204, #9106 S), phosphorylated SAPK/JNK (Thr183/Tyr185, #9255 S), phosphorylated Akt (Ser473, #9271) and cleaved poly ADP ribose polymerase (PARP, Asp214, #9548 S) (all from Cell Signaling, Danvers, MA). Blots were visualized with Odyssey Infrared Imaging System (LI-COR Biosciences, Lincoln, NE).

### F-actin/G-actin assay

F- and G-actin were analyzed by homogenization of macrophages in F-actin stabilization buffer, followed by centrifugation to separate the F-actin from G-actin pools, SDS-PAGE and Western blot according to ref. 45. For details, see Supporting Information.

### Drug responsive target stability (DARTS)

F-actin and G-actin fractions (300 ng/μl) were supplemented with TNC buffer (50 mM Tris pH 8.0, 50 mM NaCl, 10 mM CaCl_2_), split into 50 μl aliquots (100 μg protein/ml) and pre-treated (30 min, 37 °C) with test compounds or 1% DMSO, respectively. Pre-treated protein samples were digested with pronase (100 ng/ml, corresponding to 1:1000 pronase/protein ratio) for 30 min at 37 °C. Digestion was stopped by addition of 2 × SDS-PAGE sample loading buffer and heated to 95 °C for 5 min. Digested protein samples were separated by SDS-PAGE and transferred to nitrocellulose membranes (Hybond-C extra). Membranes were incubated with β-actin antibody and goat anti-rabbit secondary antibody (LI-COR Biosciences). Proteins were analyzed with the Odyssey Infrared Imaging System and respective bands were normalized to signals of matching undigested controls.

### Total internal reflection fluorescence (TIRF) microscopy

Nucleation of actin in a cell-free assay was visualized by TIRF microscopy. The Actin-toolkit TIRM containing Atto488-labelled actin (Hypermol, Bielefeld, Germany) was used according to the manufacturer´s instructions; see Supporting information.

### Caspase activation assay

Caspase-3/7 activation was assessed with Apo-ONE Homogenous Caspase-3/7 Assay (Promega, Madison, WI). Macrophages (2.5 × 10^4^ cells/ml RPMI 1640, 5% FCS) were seeded in black 96-well plates and let to adhere for 1 hour. After incubation with test compounds the manufactures instructions were followed and fluorescence was measured (excitation 485 nm, emission 530 nm, Novostar, BMG Labtechnologies, Offenburg, Germany).

### Cytokine and chemokine assays

Cytokine and chemokine levels were determined by sandwich ELISA using the DuoSet Kit from R&D Systems (Minneapolis); see Supporting information.

### NFκB activation (p65) by fluorescence microscopy

Macrophages were adhered onto coverslips, treated with test compounds or 0.05% DMSO for 30 min, followed by stimulation with either 100 ng/ml LPS and 100 ng/ml IFN-γ (M1) or with 20 ng/ml IL-4 (M2). After 30 min, cells were fixed with 4% paraformaldehyde, permeabilized with 100% acetone and after washing incubated with 0.25% Tween 20. After blocking with 10% non-immune goat serum, incubation with primary antibodies against p65 over night at 4 °C, and incubation with secondary antibodies, cells were incubated with DAPI, and the fluorescence was visualized with a Zeiss Axio Observer. Z1 microscope, see above.

### Determination of reactive oxygen species (ROS) formation

The peroxide-sensitive dye 2′,7′-dichlorofluorescin-diacetate (1 μg/ml, Sigma-Aldrich) was added to macrophages (10^6^/ml in PBS containing 0.1% glucose and 1 mM CaCl_2_) in a black 96 well-plate (Greiner bio-one). The reaction was started by addition of the test compounds. The fluorescence emission (520 nm) was measured after excitation at 485 nm in a spectrofluorometer (Novostar, BMG Labtech, Ortenberg, Germany) for 250 min at 37 °C.

### Statistics

Results are expressed as mean ± standard error of the mean (SEM) of *n* observations, where *n* represents the number of experiments performed independently. Statistical evaluation of the data was performed by ANOVA for independent or correlated samples followed by Bonferroni *post-hoc* tests. Where appropriate, Student’s t test was applied. The tests were conducted using a two-sided alpha level of 0.05 (*p < 0.05).

## Additional Information

**How to cite this article**: Pergola, C. *et al*. Modulation of actin dynamics as potential macrophage subtype-targeting anti-tumour strategy. *Sci. Rep.* **7**, 41434; doi: 10.1038/srep41434 (2017).

**Publisher's note:** Springer Nature remains neutral with regard to jurisdictional claims in published maps and institutional affiliations.

## Supplementary Material

Supporting Information
