# Supplementary material for: Modulation of actin dynamics as potential macrophage subtype-targeting anti-tumour strategy
Source: Sci Rep. 2017 Jan 30;7:41434. doi: 10.1038/srep41434 (PMC5278352; doi:10.1038/srep41434)
Supplement: Supporting Information [file srep41434-s1.pdf]

Supplementary Information

Modulation of actin dynamics as potential macrophage subtype-targeting anti-tumour strategy

Carlo Pergola, Katrin Schubert, Simona Pace, Jana Ziereisen, Felix Nikels, Olga Scherer, Stephan Hüttel, Stefan Zahler, Angelika Vollmar, Christina Weinigel, Silke Rummler, Rolf Müller, Martin Raasch, Alexander Mosig, Andreas Koeberle, Oliver Werz

Contents:

Detailed description of MTT assay
Detailed description of F-actin/G-actin assay
Detailed description of monocyte migration assay
Detailed description of fluorescence microscopy
Detailed description of microfluidically supported biochips assay: preparation of biochips
Detailed description of Western Blot
Detailed description of cytokine and chemokine assays
Detailed description of total internal reflection fluorescence (TIRF) microscopy
Supplementary Figure 1
Supplementary Figure 2
Supplementary Figure 3
Supplementary Figure 4
Supplementary Figure 5
Supplementary Figure 6
Supplementary Figure 7
Supplementary Figure 8

MTT Assay

Macrophages, LNCaP or MDA-MB-231 were treated with vehicle (0.05% DMSO) or ChA at 37 °C. After the indicated times, 20 µl of 3-(4,5-dimethylthiazol-2-yl)-2,5-diphenyltetrazolium bromide (MTT) solution (5 mg/ml in PBS, Sigma-Aldrich) were added and incubated for 3 h at 37 °C. Afterwards, 100 µl MTT-lysis buffer (10% SDS in 20 mM HCl, pH 4.5) were added for 18 h at room temperature (RT), and the absorbance was measured at 570 nm (Multiskan Spectrum, Thermo Fisher Scientific, Vantaa, Finland).

F-actin/G-actin assay

Macrophages (1 × 10^6 cells/ml RPMI 1640, 5% FCS) were allowed to adhere for 1 h onto glass coverslips (Roth, Karlsruhe, Germany) in a 12-well plate and then incubated at 37 °C with vehicle (0.1% DMSO) or test compounds. Cells were then collected and lysed with 100 µl of pre-warmed lysis and F-actin stabilization buffer (50 mM PIPES pH 6.9, 50 mM NaCl, 5 mM $MgCl_2$,5 mM EGTA, 5% (v/v) glycerol, 0.1% (v/v) Nonidet P40, 0.1% (v/v) Triton X-100, 0.1% (v/v) Tween 20, 0.1% (v/v) β-mercaptoethanol, 0.001% (v/v) Antifoam C plus freshly added 10 µl/ml of 100 mM ATP, and 10 µl/ml Protease inhibitor cocktail). Cells were homogenized using a 200 µl pipette tip with a fine orifice (up and down, eight times) and incubation at 37 °C for 10 min. Cell lysis was checked by trypan blue staining and light microscopy. The lysate was centrifuged (600 × *g,* 5 min, 4 °C) to remove unbroken cells, and the homogenate was then centrifuged at 100,000 × *g* for 60 min at 4 °C. The resulting supernatants were immediately put on ice, the pellets were resuspended with ice cold Milli-Q water plus 10 µM cytochalasin D and left on ice for 1 h to dissociate F-actin, and pipetted once

every 15 min with a fine 200 µl pipette tip. Samples were then mixed with 4×SDS-PAGE sample loading buffer, heated to 95°C for 5 min and analyzed by SDS-PAGE and Western blot.

Monocyte migration assay

Monocyte migration in response to MCP-1 (100 ng/ml, PeproTech) was evaluated using the microplate system ChemoTx® (Neuro Probe, Inc., Gaithersburg, MD), where microchambers were separated from the upper compartment by polycarbonate membranes (pore size 5 µm). Human recombinant MCP-1 (100 ng/ml, PeproTech) in RPMI 1640 medium was added to the lower compartment of the disposable 96-well chemotaxis chamber in a total volume of 29 µl. Resuspended monocytes (1×10^5 cells/well in 25 µl) were added to the upper compartment of the chamber that had been pre-coated with FCS for 2 h and incubated at 37°C and 5% CO_2 for another 2 h. Then, the filter was removed and the number of migrated monocytes was evaluated by ATP analysis. ATP was analyzed according to the manufacturer´s protocol (CellTiter-Glo Luminescent Cell Viability Assay, Promega, Madison, WI). Luminescence was measured (Novostar, BMG Labtechnologies, Offenburg, Germany) and the values were compared to a simultaneously obtained standard curve of monocytes from a range of zero to 1×10^5 cells.

Fluorescence microscopy

Macrophages (2.5×10^5 cells/ml RPMI 1640, 5% FCS) were allowed to adhere for 1 h onto Petri dishes with glass bottom (MatTek Corporation, Ashland, MA) or glass coverslips (Roth, Karlsruhe, Germany) in a 12-well plate and then incubated at 37 °C with vehicle (0.1% DMSO) or test compounds. Cells were incubated with a fluorescent BODIPY-chondramide derivative (Suppl. Fig. 3A, generous gift from Prof. Dr. H.-D. Arndt, University Jena), and life-cell imaging was performed. Alternatively, cells were fixed in 4% formaldehyde in PBS (20 min, RT) and permeabilized with 0.2% Triton X-100 (5 min, RT). The staining was performed with

Alexa Fluor 488 phalloidin (5 units/ml PBS + 1% BSA, Invitrogen, Oregon) and Alexa Fluor 594 DNase I conjugates (9 µg/ml PBS + 1% BSA, Invitrogen) for 20 min at RT in the dark. DNA was stained with 0.6 µg/ml 4',6-diamidino-2-phenylindole dihydrochloride (DAPI, Sigma-Aldrich) for 3 min at RT in the dark. Coverslips were mounted on glass slides (Roth) with polyvinylalcohol 4-88 (Sigma-Aldrich) containing 0.25% *n*-propyl gallate (Sigma-Aldrich). The fluorescence was visualized with a Zeiss Axio Observer.Z1 microscope and a LCI Plan-Neofluar 63x/1.3 Imm Corr DIC M27 Objective (Carl Zeiss AG, Jena, Germany). Images were taken with an AxioCam MR3 camera and were acquired, cut, linearly adjusted in the overall brightness and contrast, and exported to TIF by the AxioVision 4.8 software.

Microfluidically supported biochips assay: preparation of biochips

Biochips were made by injection moulding from cyclo olefin polymer (COP) Zeonor®, obtained from microfluidic ChipShop GmbH (Jena, Germany), and manufactured as described previously [1]. Briefly, a 12.2 µm thick PET membrane with a pore diameter of 8 µm and a pore density of 1 × 10^5 pores/cm² (Sabeu, Radeberg, Germany) was integrated. Chips and channel structures were sealed on top and bottom side with an extruded 140 µm thick COP foil using a low temperature proprietary bonding method. Oxygen plasma treatment for hydrophilisation of the whole chip surface was performed to support cell growth and to reduce in-chip air bubble formation. Additionally, equilibration of the medium under perfusion conditions was performed to reduce air bubble formation. Gas permeable silicon tubing was used for perfusion allowing oxygen equilibration during experiments.

In each sterilized biochip 1.3×10^5 HUVEC/cm² and 0.43×10^5 monocytes / cm² were mixed and cultured for 72 h in ECGM supplemented with 10% autologous serum and Pen/Strep. 10 ng/ml M-CSF was added for macrophage differentiation. On day four, 1.3×10^5 MCF-7 cells/cm² were

seeded in the lower compartment and cultured for two more days in DMEM-HG plus 10% FCS. Macrophage polarization was induced on day five by replacing the medium with ECGM plus 10% autologous serum, 10 ng/ml M-CSF, Pen/Strep and 10 ng/ml IFN-γ for M1 or 10 ng/ml IL-4 for M2 polarization for 24 h. Medium containing 1 µM ChA or vehicle was perfused over the vascular layer with a shear stress rate of 3 dyn/cm² for 2 h. Medium was replaced with ECGM plus 10% autologous serum, 10 ng/ml M-CSF, Pen/Strep and 10 ng/ml IFN-γ for tumours models using M1 or 10 ng/ml IL-4 for M2; tumour models were cultured for additional 48 h. Where indicated, 1 µg/ml TNFα capture antibody (R&D systems, MN) was added. For subsequent analysis, supernatants were collected on a daily basis.

For Calcein-AM staining, HUVEC and MCF-7 layers were incubated (15 min) with 2.5 µM Calcein-AM (LifeTechnologies, Carlsbad, CA) and subsequently analyzed by life cell fluorescence microscopy. For immunostaining, HUVEC were fixed with 2% paraformaldehyde and stained with antibodies against VE-Cadherin (BD Bioscience/Pharmingen, Heidelberg, Germany) and a secondary goat-anti-mouse-Cy3 (Dianova, Hamburg, Germany) antibody. Samples were embedded into fluorescent mounting medium (Dako, Carpinteria, CA). Imaging was performed on an Axio Observer Z.1 with AxioVision 4.8.3 software (Carl Zeiss AG). Images were analyzed using ImageJ2/Fiji.

After perfusion, the medium within the lower chamber containing MCF-7 cells was collected, and cytokines were measured using a multiplexed bead-based immunoassay (Cytometric bead array (CBA), BD Bioscience/Pharmingen, Heidelberg, Germany). Cytokine kits used: human enhanced sensitivity TNFα Flex Set, human enhanced sensitivity IL-1β Flex Set, human IL-6 Flex Set, human IL-8 Flex Set and human IL-10 Flex Set (BD Bioscience/Pharmingen). Analysis was performed with a FACS Canto II flow cytometer (BD Bioscience/Pharmingen) and data were quantified using FlowJo v10 software (TreeStar, Ashland OR).

Western Blot

Macrophages were treated with compounds and lysed (TBS, pH 7.4, 1% NP-40, 1 mM Na_3VO_4, 10 mM NaF, 5 mM sodium pyrophosphate, 25 mM β-glycerophosphate, 5 mM EDTA, 10 µg/ml leupeptin, 60 µg/ml STI, 1 mM PMSF) for 30 min on ice. Samples were centrifuged (10,000×*g*, 5 min, 4 °C) and the supernatant was mixed with 4×SDS-PAGE sample loading buffer (200 mM Tris-HCl, pH 6.8, 8% SDS, 40% glycerol, 4% β-mercaptoethanol, 50 mM EDTA, 0.08% bromophenol blue), heated to 95°C for 5 min, and analyzed by SDS-PAGE on a 10% gel followed by protein transfer onto nitrocellulose membrane (Hybond-C extra, Amersham BioSciences, Buckinghamshire, UK).

Cytokine and chemokine assays

Cytokine and chemokine levels were determined by sandwich ELISA using the DuoSet Kit from R&D Systems (Minneapolis). Maxisorp Immunomodules (Nunc, Roskilde, Denmark) were coated with the primary antibody over night at 4 °C. After washing with PBS containing 0.05% Tween 20 the plate was blocked with 1% BSA in PBS for 1 hour at RT. The cell culture supernatants were added to the plate and incubated for 2 h. Then, the secondary antibody was added for additional 2 h followed by 30 min incubation of Streptavidin-HRP. Afterwards, 1-step Ultra-TMB-ELISA (Thermo Scientific) was added for 30 min and the reaction was terminated by addition of 2 M H_2SO_4. The absorbance was measured at 450 nm (Multiskan Spektrum).

Total internal reflection fluorescence (TIRF) microscopy

Nucleation of actin in a cell-free assay was visualized by TIRF microscopy. The Actin-toolkit TIRM containing Atto488-labelled actin (Hypermol, Bielefeld, Germany) was used according to the manufacturer´s instructions. Coverslips were coated with 0.1 mg/ml NEM-myosin in order to tether actin nuclei or filaments. Actin polymerization in a 1 µM solution was started by adding a respective high salt buffer. Due to sample handling and focusing on the TIRF microscope there is a delay of approx. 30 seconds. Images were obtained on a Leica TIRF MC microscope equipped with a 100× oil immersion TIRF lens at an excitation of 488 nm and an emission of 505 – 520 nm every 10 seconds for 15 minutes.

Supplementary Figures

Suppl. Fig. 1

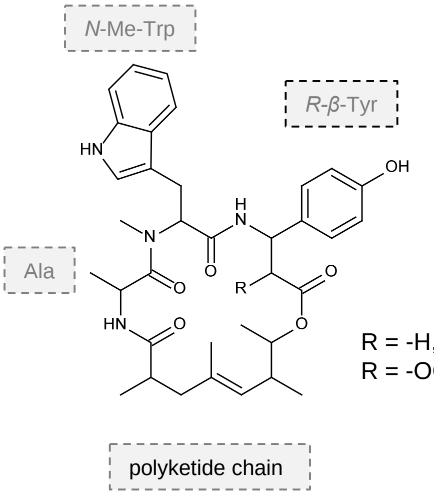

R = -H, chondramide C
R = $-OCH_3$, chondramide A

Suppl. Figure 1. Chemical structure of chondramide A and C.

Suppl. Fig. 2

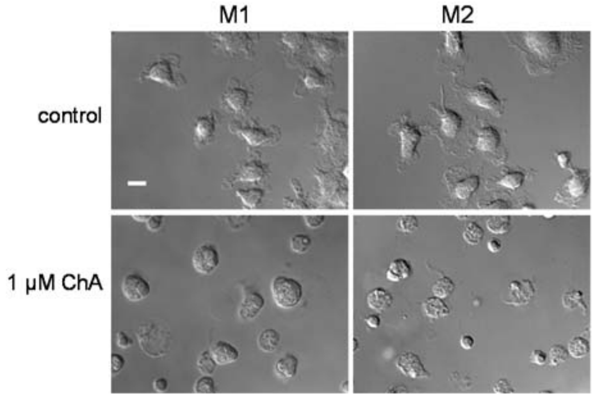

Suppl. Figure 2. Lytic cell death of M2 after ChA incubation. M1 and M2 cell morphology and integrity after incubation with vehicle (0.1% DMSO) or 1 µM ChA for 24 h were visualized by phase contrast microscopy of living cells; n = 3; scale bar, 10 µm.

Suppl. Fig. 3

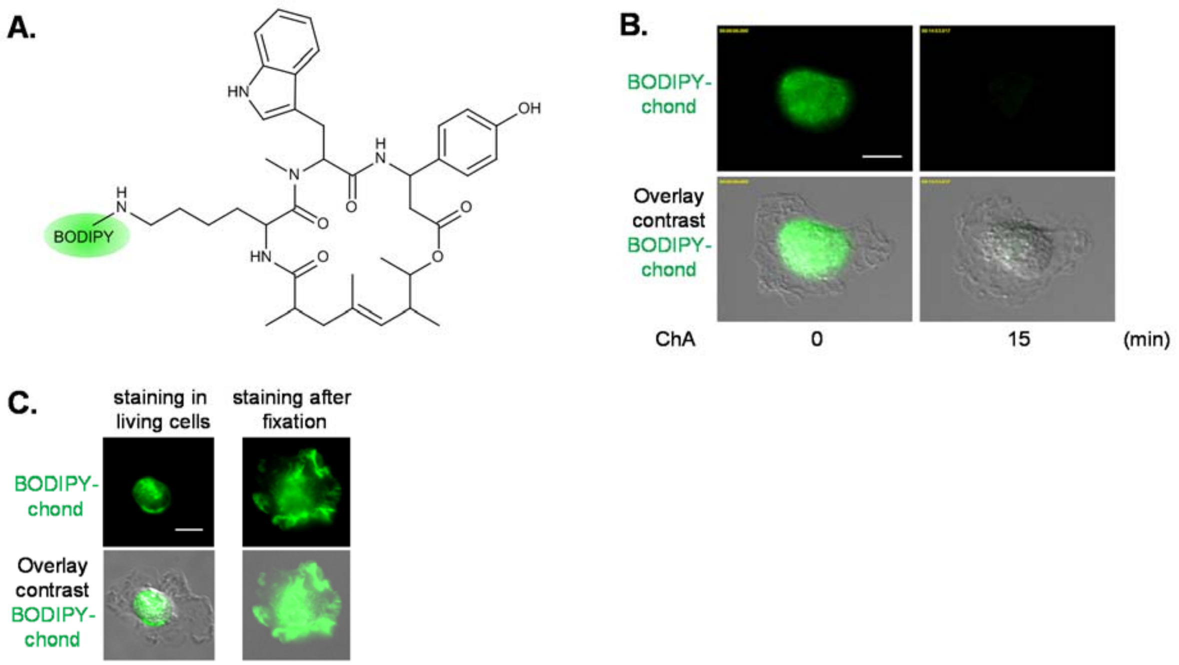

Suppl. Figure 3. Live cell imaging with fluorescent BODIPY-modified chondramide. (A) Structure of the cell permeable BODIPY-modified chondramide derivative used. (B,C) Live cell imaging of macrophages with fluorescent BODIPY-modified chondramide probe (1 µM, green), followed by (B) competition with 1 µM ChA for 15 min. (C) Staining in living cells and after cell fixation. The pictures shown are representative of three independent experiments.

Suppl. Fig. 4

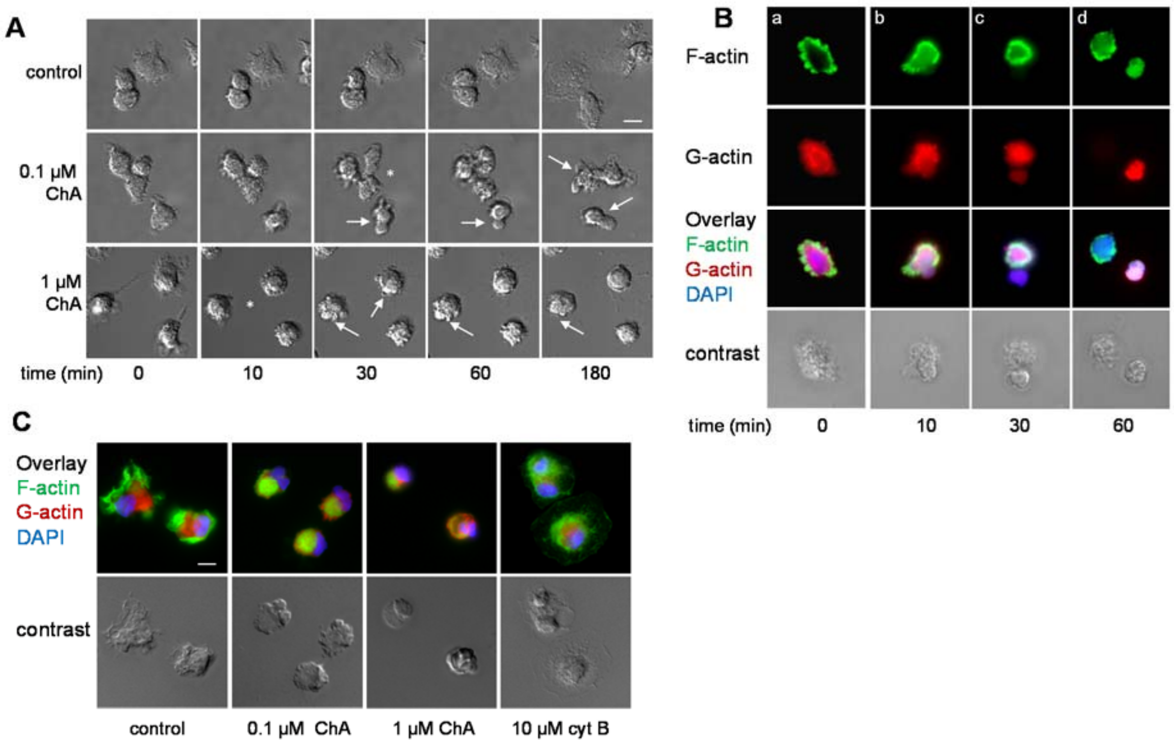

Suppl. Figure 4. Effect of ChA on actin in living cells. (A) Phase contrast microscopy of living M0 after treatment with vehicle (0.1% DMSO) or the indicated concentrations of ChA for the indicated times. Stars indicate cell contraction and loss of lamellar processes, arrows indicate budding events. (B,C) Analysis of F- and G-actin localization in M0 after treatment with (B) 1 µM ChA for the indicated times or (C) vehicle control (0.1% DMSO), 0.1 µM or 1 µM ChA, or 10 µM cytochalasin B (cyt B) for 24 h. Staining was performed after cell fixation with Alexa Fluor 488 phalloidin (green, F-actin), Alexa Fluor 594 DNase I (red, G-actin), and DAPI (blue, nuclei); scale bar, 10 µm. The pictures shown are representative of three independent experiments.

Suppl. Fig. 5

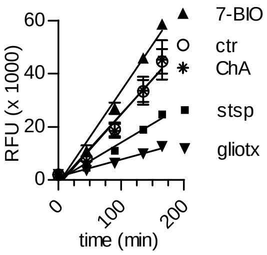

Suppl. Figure 5. ChA does not induce ROS formation. Spontaneous ROS formation in macrophages (measured by 2',7'-dichlorofluorescin-diacetate) is not increased by ChA treatment. The compound 7-bromo indirubin 3′-O-monoxime (7-BIO, 1 µM; generous gift of Prof. Dr. Leandros Skaltsounis, University of Athens), staurosporine (stsp, 1 µM) and gliotoxin (gliotox, 1 µM) were used as positive controls. Data are given as means + SEM; n = 3.

Suppl. Fig. 6

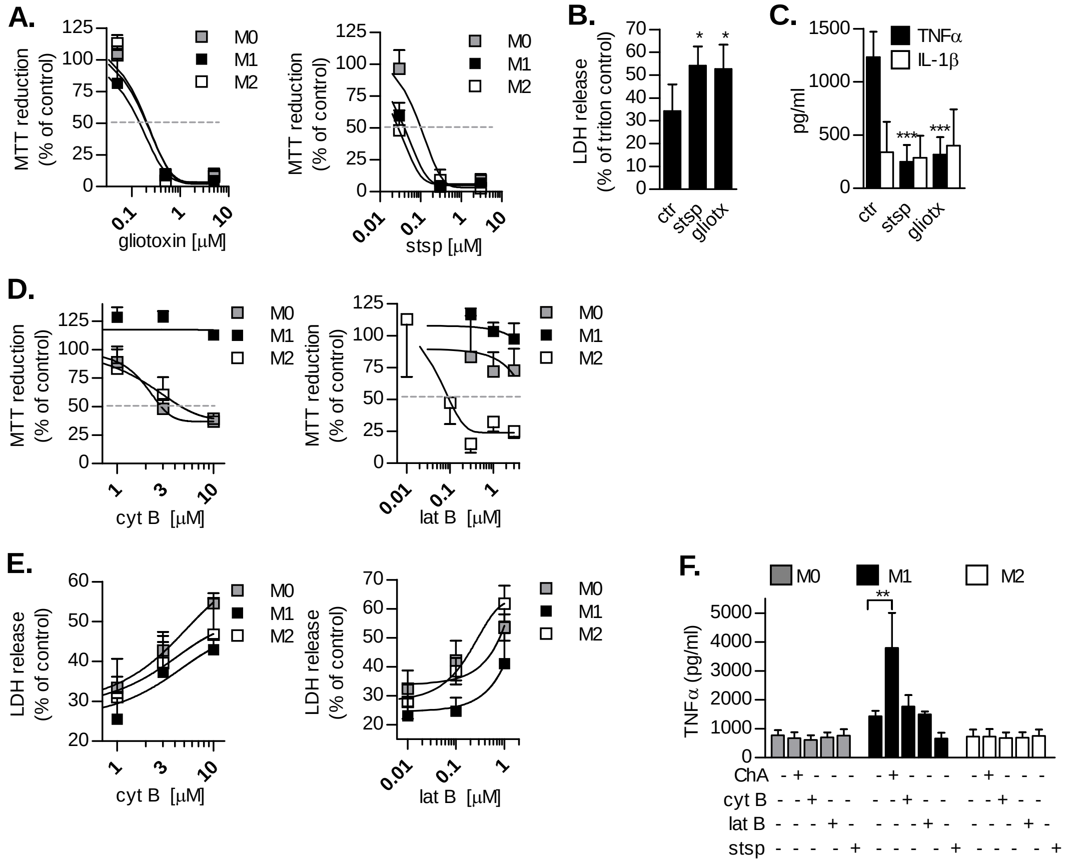

Suppl. Figure 6. Macrophage toxicity of anti-tumoural or actin-interfering agents and induction of TNFα and IL-1β release. (A,D) MTT and (B,E) LDH assay or (C,F) TNFα and (C) IL-1β release for analysis of metabolic activity, membrane integrity, and cytokine release, respectively, of (A,B,D-F) M0, (A,C,D-F) M1, and (A,D,E,F) M2 after incubation with gliotoxin, staurosporine (stsp), cytochalasin b (cyt B) or latrunculin B (lat B) for 48 h (MTT) or 24 h (LDH). In (B,C), gliotoxin (gliotx) and staurosporine (stsp) were used at 1 µM. In (F) ChA, staurosporine (stsp) and latrunculin B (lat B) were used at 1 µM, while cytochalasin B (cyt B) was used at 10 µM. Compounds were added 30 min before the polarization or corresponding incubation without stimuli (for M0) were started. Data are expressed as percentage of (A,D) vehicle (0.1% DMSO) or (B,E) Triton X-100 (1%, resulting in total membrane disruption) controls or as (C,F) absolute values and are given as means + SEM; n = 3; *$p < 0.05$, **$p < 0.01$, ***$p < 0.001$ vs vehicle control; ANOVA + Bonferroni.

Suppl. Fig. 7

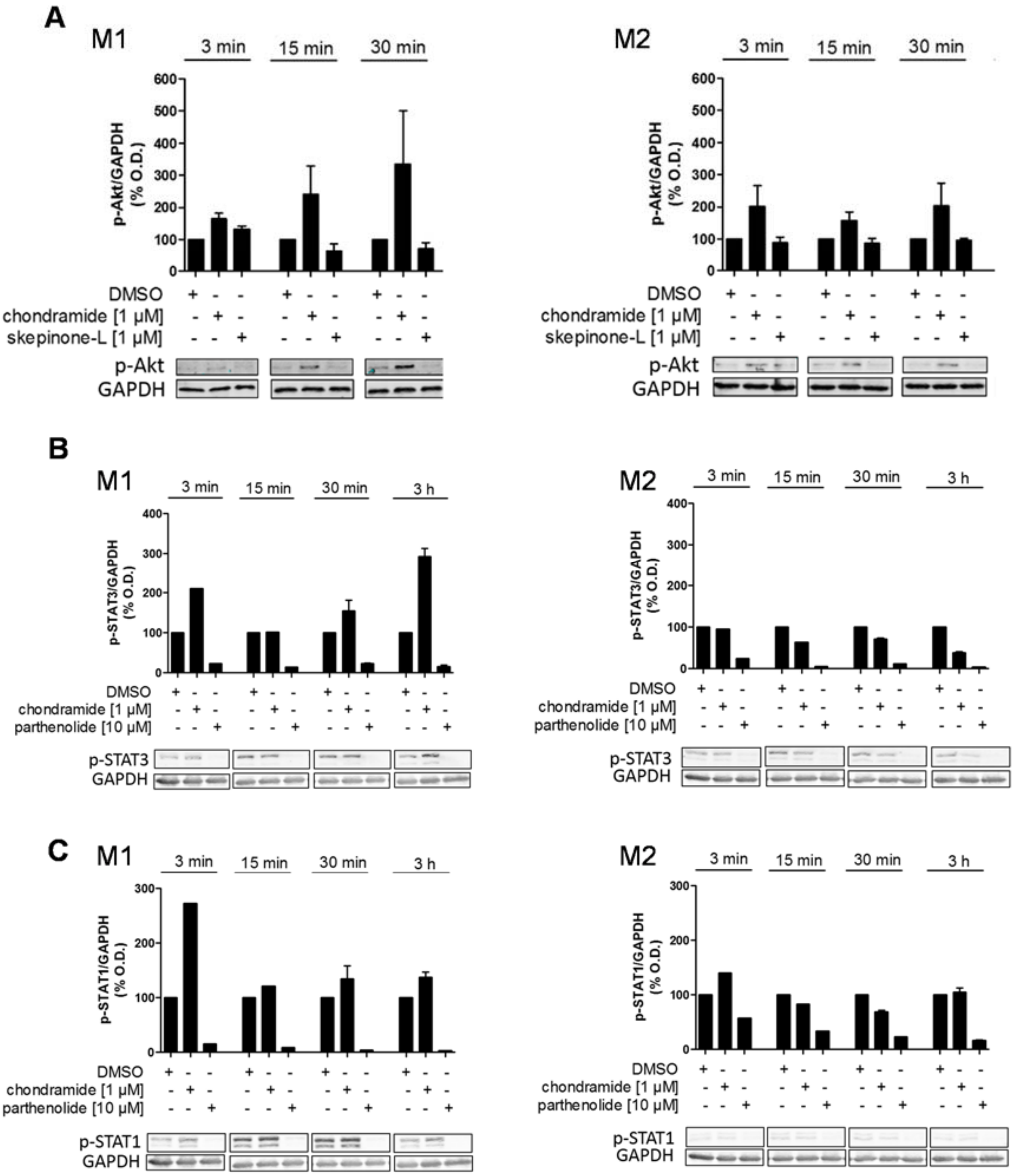
A
M1
3 min
15 min
30 min
p-Akt/GAPDH (% O.D.)
DMSO
chondramide [1 µM]
skepinone-L [1 µM]
p-Akt
GAPDH
M2
B
p-STAT3/GAPDH (% O.D.)
3 h
parthenolide [10 µM]
p-STAT3
C
p-STAT1/GAPDH (% O.D.)
p-STAT1

Suppl. Fig. 7

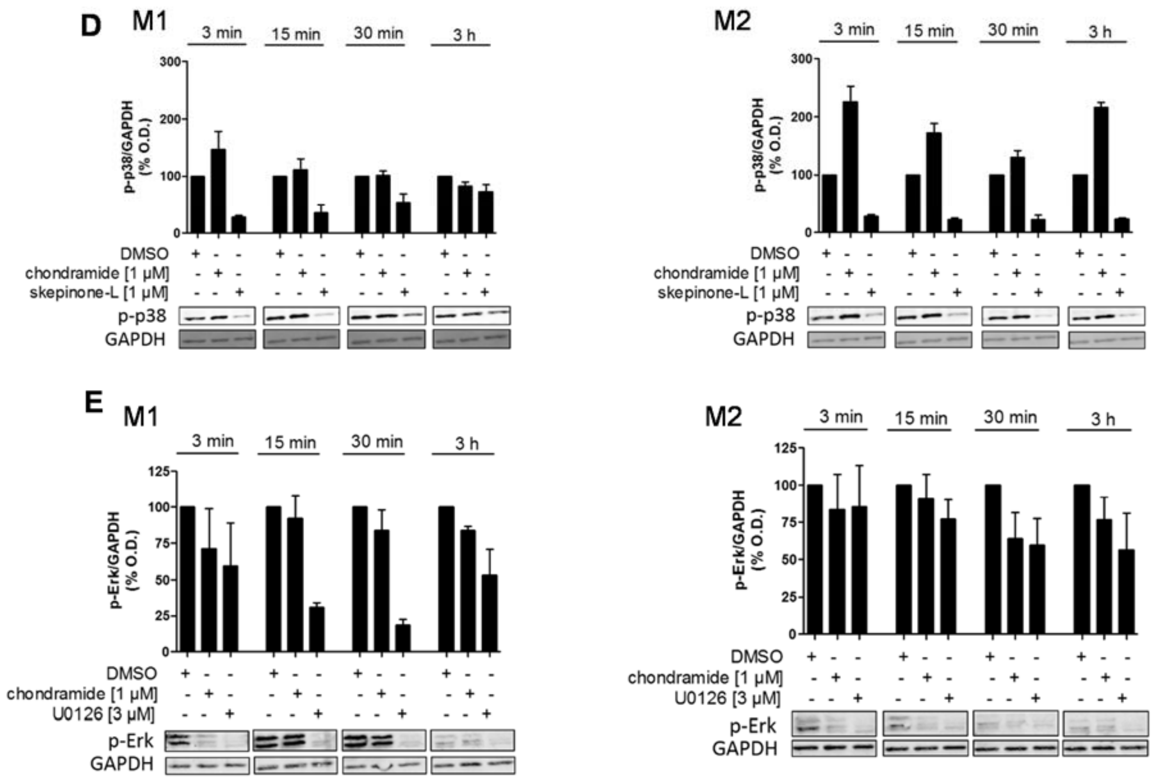

Suppl. Figure 7. **Induction of protein kinase phosphorylation by ChA in M1 and M2.** Macrophages were pre-treated with 1 µM ChA, 10 µM parthenolide, 1 µM skepinone-L, or vehicle (0.5% DMSO) for 30 min. Then, LPS/INFγ to obtain M1 (left panels) or IL-4 to obtain M2 (right panels) was added, as indicated. Cells were lysed after the indicated time points and the amounts of phosphorylated Akt (A), phosphorylated STAT-3 (B), phosphorylated STAT-1 (C), phosphorylated p38 MAPK (D), and phosphorylated ERK-1/2 (E) were analyzed by Western blot. GAPDH was used for normalization of protein contents of the samples. Densitometric data (normalized to GAPDH) are expressed as percentage of vehicle control and are given as means + SEM; n = 3.

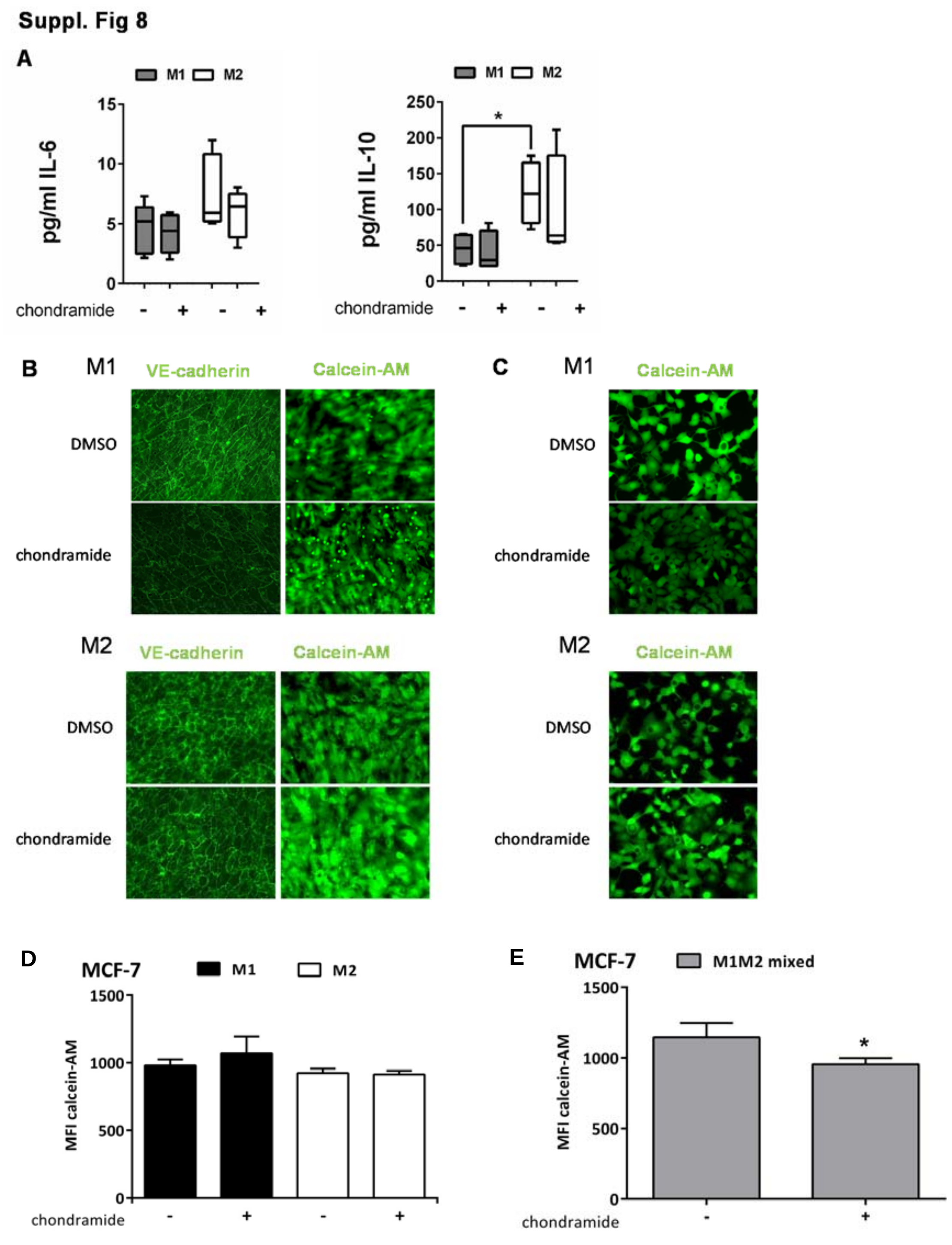

Suppl. Figure 8. Effects of ChA on cytokine release, VE cadherin in HUVECs and MCF-7 cell viability in a biochip-based tumour model. (A) Release of IL-6 and IL-10 upon 1 µM ChA treatment in the biochip-based tumour models composed of M1 or M2. Data are expressed as means + SEM; n = 3; *$p < 0.05$; ANOVA + Bonferroni. (B,C) Biochip-based tumour models

containing M1 or M2 were treated with ChA (chondramide) or vehicle control (0.1% DMSO). (B) Fluorescence images of the endothelial layer stained for VE cadherin for analysis of vascular barrier function or with Calcein-AM for analysis of viability. (C) Fluorescence images of MCF-7 cells stained with Calcein-AM. Representative images of three independent experiments are shown. (D, E) Viability of MCF-7 cells by Calcein-AM staining after 48 h incubation of HUVEC (D) without macrophages, in the presence 1 μM ChA or vehicle (0.1% DMSO) under M1 or M2 polarization conditions, or (E) with a mixture of M1 and M2 (1:1), each. Mean fluorescence intensity (MFI) was measured by random field analysis of 30 regions of interest per experiment. Data, means + SEM, n=3. *, $p < 0.05$.

Literature

[1] Raasch M et al. Microfluidically supported biochip design for culture of endothelial cell layers with improved perfusion conditions. *Biofabrication* **7**, 5013 (2015)
